# ﻿*Pinctadaphuketensis* sp. nov. (Bivalvia, Ostreida, Margaritidae), a new pearl oyster species from Phuket, western coast of Thailand

**DOI:** 10.3897/zookeys.1119.87724

**Published:** 2022-09-02

**Authors:** Supannee Somrup, Akkarasiri Sangsawang, Nichanun McMillan, Supanida Winitchai, Jitti Inthoncharoen, Shikai Liu, Narongrit Muangmai

**Affiliations:** 1 Key Laboratory of Mariculture, Ministry of Education, Ocean University of China, Qingdao 266003, China; 2 Department of Aquaculture, Faculty of Fisheries, Kasetsart University, Bangkok 10900, Thailand; 3 Kasetsart Agricultural and Agro-Industrial Product Improvement Institute (KAPI), Kasetsart University, Bangkok 10900, Thailand; 4 Phuket Pearl Industry, Co. Ltd., Phuket 83000, Thailand; 5 Department of Fishery Biology, Faculty of Fisheries, Kasetsart University, Bangkok 10900, Thailand; 6 Animal Genomics and Bioresource Research Center (AGB Research Center), Faculty of Science, Kasetsart University, Bangkok 10900, Thailand; 7 Biodiversity Center Kasetsart University (BDCKU), Bangkok 10900, Thailand

**Keywords:** Indian Ocean, mollusk, new species, phylogeny, taxonomy

## Abstract

A new species of the genus *Pinctada* is described from samples collected from the east coast of Phuket Island, Thailand in the Andaman Sea. *Pinctadaphuketensis***sp. nov.** is distinguished from other species on both molecular and morphological data. Morphologically, the valves of *P.phuketensis* are characterized by a slightly developed to undeveloped posterior auricle, a small, narrow slit-like byssal notch, the absence of hinge teeth, and a pale to transparent non-nacreous border, with a few dark brown or red blotches. This new species resembles *P.fucata* but differs by its smaller size and the absence of hinge teeth. Phylogenetic analyses based on both mitochondrial (COI) and nuclear (18S rDNA, ITS1 and ITS2) genes and species delimitation using COI data strongly support that *P.phuketensis* is a distinct species, which is closely related to *Pinctadaalbina* and *Pinctadanigra*.

## ﻿Introduction

Pearl oysters in the genus *Pinctada* (Röding, 1798) (family Pteriidae) are widely distributed from shallow to deep waters of the tropical and subtropical regions between the Indo-Pacific and western Atlantic (Wada and Tëmkin 2008; Cunha et al. 2011). *Pinctada* contains approximately 20 species according to the latest taxonomic records of MolluscaBase (2022). Several *Pinctada* species are used widely in pearl aquaculture and in industry including the Akoya pearl oyster *Pinctadafucata* (Gould, 1850) in Japan (Matsuyama et al. 2021); the black-lip pearl oyster *Pinctadamargaritifera* (Linnaeus, 1758) in the South Pacific and Indo-Pacific Islands (Aideed et al. 2014; Ky et al. 2019); and the silver-lipped pearl oyster, *Pinctadamaxima* (Jameson, 1901) in western Australia (Steve et al. 2021).

While the pearl farming industry has expanded rapidly during recent decades, our understanding of biodiversity, evolution, and conservation of *Pinctada* species is still limited. Traditionally, systematics and taxonomy of *Pinctada* species have primarily focused on morphological parameters (Hynd 1955, 1960; Wada and Tëmkin 2008). The identification of *Pinctada* species is largely based on the soft tissues and shell characteristics; however, such morphological features vary greatly and are sometimes indistinguishable between species, particularly if the specimens are young (Ranson 1961; Wada and Tëmkin 2008). Accordingly, these studies are relatively complicated and challenging due either to their non-discrete differentiation or high levels of morphological variation (Cunha et al. 2011; Scuderi et al. 2019). In order to address these problems related to morphology-based taxonomy, molecular approaches, together with detailed comparative morphology have been increasingly applied to elucidate the classification, distribution pattern and evolutionary history of *Pinctada* species (Yu and Chu 2006; Tëmkin 2010; Cunha et al. 2011; Lal et al. 2018; Reisser et al. 2019). Additionally, a recent proposal to raise the infraspecific taxon *P.margaritiferapersica* to specific rank as *P.persica* (Jameson) has been supported primarily by partial mitochondrial cytochrome oxidase subunit 1 (COI) sequences and two different species delimitation methods (general mixed Yule-coalescent: GMYC and Automatic Barcode Gap Discovery: ABGD) (Sharif Ranjbar et al. 2016). The aforementioned study clearly confirms the potential of DNA sequences to unveil hidden diversity, geographic origin, and phenotypic plasticity of pearl oyster *Pinctada* species.

In the Southeast Asian region, nine species of *Pinctada* are currently recognized: *P.albina* (Lamarck, 1819), *P.chemnitzii* (Phillipi, 1849), *P.fucata*, *P.imbricata* Röding, 1798, *P.maculata* (Gould, 1850), *P.margaritifera*, *P.maxima*, *P.nigra* (Gould, 1850) and *P.radiata* (Leach, 1814) (Cheah 2007; Sanpanich and Duangdee 2018; MolluscaBase 2022). All nine *Pinctada* species have been recorded in Thailand (Wells et al. 2021). However, diversity and taxonomic studies of Thai *Pinctada* species have relied heavily on morphological features, and the research is outdated when compared with studies from other areas, such as the Central Pacific Ocean (Yu et al. 2006; Miyake et al. 2016; Saruwatari et al. 2018) and Indo-West Pacific (Colgan and Ponder 2002; Cunha et al. 2011; Reisser et al. 2019). Considering this fact, we postulate that the diversity of *Pinctada* species has yet to be fully revealed in Thai waters and adjacent areas.

Among Thai species, *P.fucata* and *P.maxima* are the main species used for pearl culture in Phuket, the island province off the western coast of Thailand (Bussarawit 1995; Kanjanachatree et al. 2004). As a consequence of great abundance and high demand for these two *Pinctada* species, most previous studies focused on their life cycle, physiology and cultivation techniques (Kanjanachatree et al. 2004, 2006; Piyathamrongrut et al. 2009), whereas little is known about their biodiversity and genetic resources. We recently collected several *Pinctada* specimens from Phuket, and some of them were quite different in external appearance from other reported *Pinctada* species in this area. Accordingly, the present study aims to clarify the taxonomic status of these recently collected *Pinctada* specimens based on morphological and molecular analyses.

## ﻿Materials and methods

A total of 15 pearl oyster specimens were collected around Dok Mai Island (7°47.84'N, 98°31.84'E), Phuket Province, western coast of Thailand by SCUBA diving. All specimens were allocated a registration code (NMR) to facilitate sample tracking. A small piece of adductor muscle from each oyster was preserved in 90% ethanol for DNA analyses. For morphological observation, we carefully examined both shell and soft body features (Wada and Tëmkin 2008), especially shell shape, hinge teeth pattern, posterior auricle and byssal notch. All characteristics were observed under the stereomicroscope. Voucher specimens were deposited at Kasetsart University Museum of Fisheries (Natural History Museum) mollusk collection (KUMF.MOLL.), Faculty of Fisheries, Kasetsart University, Thailand.

Genomic DNA extraction from mantle tissue was performed using NucleoSpin Tissue Kit (Macherey-Nagel, Germany). Mitochondrial cytochrome oxidase subunit 1 (COI) gene, nuclear 18S rDNA gene and nuclear ribosomal DNA internal transcribed spacer 1 and 2 (ITS1 and ITS2) regions were selected for molecular phylogenetic analysis according to previous studies (e.g., Yu and Chu 2006; Tëmkin 2010; Cunha et al. 2011; Sharif Ranjbar et al. 2016). Primer details, PCR amplification profile and procedure followed Folmer et al. (1994) for COI, Tëmkin (2010) for 18S rDNA and Yu and Chu (2006) for ITS1 and ITS2. PCR was carried out using PCR Master Mix solution (i-Taq^TM^) (iNtRON Biotechnology DR, South Korea) in a total volume of 20 μl, consisting of 10 μl of i-Taq, 10 pmol of each primer and 2 μl of DNA (~ 10–20 ng). PCR products were purified with ExoSAP-IT (USB, Cleveland, Ohio USA) and then sequenced commercially (U2Bio Inc., Seoul, South Korea).

Newly generated sequences, including seven COI sequences, six 18S rDNA sequences and five ITS1 and ITS2 sequences, were deposited in NCBI. All sequences were edited, assembled, and aligned for individual and concatenated data sets using the Geneious Prime software package (Biomatters, available from http://www.geneious.com/) with the MAFFT sequence alignment algorithm, and were further manually refined. Additional sequences of *Pinctada* species were retrieved from NCBI and included in the alignment (Suppl. material 1). *Pteria* (Scopoli, 1777) species were selected as outgroups.

Phylogenetic trees were reconstructed for both individual (COI and 18S rDNA) and concatenated data sets (ITS1 + ITS2) using maximum likelihood (ML) implemented in IQ-TREE (Minh et al. 2020) and Bayesian inference (BI) implemented in MrBayes v.3.2 (Ronquist et al. 2012). ML analyses were carried out with the best-fit model selection implemented in ModelFinder (Kalyaanamoorthy et al. 2017). The nodal support values were estimated using the nonparametric bootstraps with 1000 replicates. For BI analyses, the nucleotide models of substitution were selected using Kakusan 4 (Tanabe 2011). BI analyses were conducted by two parallel runs of four Markov chains for a million generations with sampling every 1000 generations. The first 2500 trees (burn-in) were removed before determining a consensus tree and posterior probabilities. The best partition schemes (partitioned by codon position for COI dataset and by gene for ITS datasets) and substitution models of ML and BI methods for all datasets are listed in Suppl. material 2. Both ML and BI trees were edited and visualized with the program FigTree v.1.4.4 (Rambaut 2019).

Additionally, due to low variation of nuclear DNA sequences among species, we utilized only COI sequences for the three different species delimitation methods: the general mixed Yule-coalescent (GMYC) model (Pons et al. 2006), the Bayesian Poisson tree processes method (bPTP) (Zhang et al. 2013) and Assemble Species by Automatic Partitioning (ASAP) (Puillandre et al. 2021). The single-threshold GMYC analyses were performed via GMYC web server (http://species.h-its.org/gmyc/) using an ultrametric input tree. Ultrametric tree was constructed using BEAST v.2.5 (Bouckaert et al. 2019) with the uncorrelated lognormal relaxed clock, the GTR + I + R model and a coalescent tree prior. For the bPTP analyses, BI tree was used as input and implemented by web server (http://species.h-its.org/ptp/) with the setting of 100,000 MCMC generations and thinning value of 100. Additionally, the ASAP approach was applied using a web server (https://bioinfo.mnhn.fr/abi/public/asap/asapweb.html). The nucleotide substitution model K2P was selected, and other parameters were set to their default values.

## ﻿Results

### ﻿Systematics

**Family Pteriidae Gray, 1847**

#### Genus *Pinctada* Röding, 1798

##### ﻿ Pinctada phuketensis

Taxon classificationAnimaliaPterioidaPteriidae

Somrup, Sangsawang, Liu & Muangmai sp. nov.

276D0C71-F257-518E-8D92-4712918FC7B7

https://zoobank.org/7B7B55A0-F42A-4067-8966-54F5B2A4ECD4

Figs 1 , 2 , 3

###### Type locality.

Dok Mai Island, Phuket Province, Thailand, 7°47.84'N, 98°31.84'E, at 5–10 m depth.

###### Material examined.

**Figure 1.**
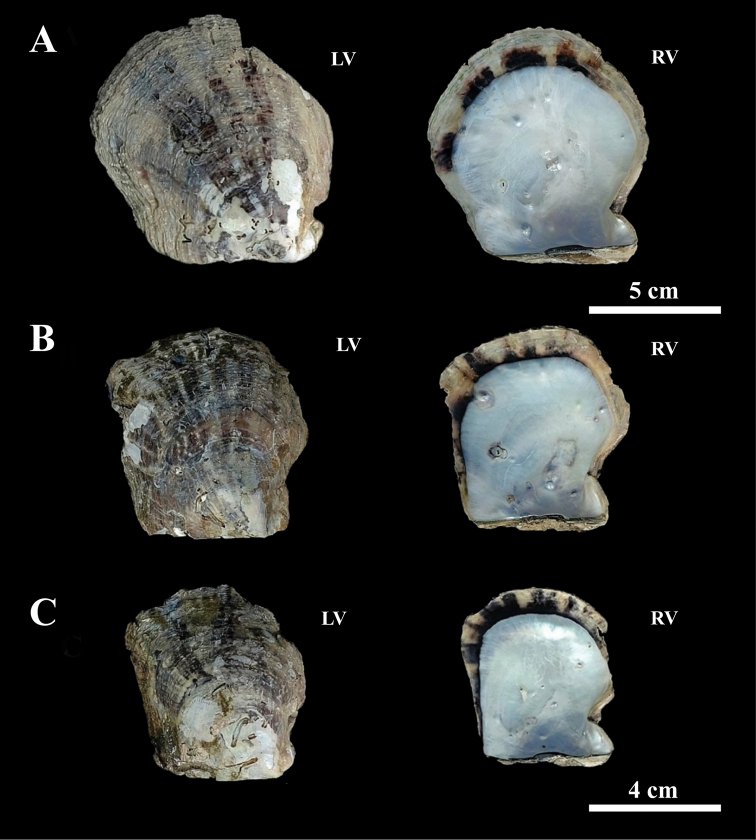
Shell of *Pinctadaphuketensis* sp. nov. from Dok Mai Island, Phuket, Thailand. External and internal views of left and right valves **A** paratype, KUMF.MOLL.1204 (NMR077) (scale bar: 5 cm) **B** holotype, KUMF.MOLL.1206 (NMRA079) **C** paratype, KUMF.MOLL.1205 (NMR078) (scale bar: 4 cm). Abbreviations: LV, left valve; RV, right valve.

**Figure 2.**
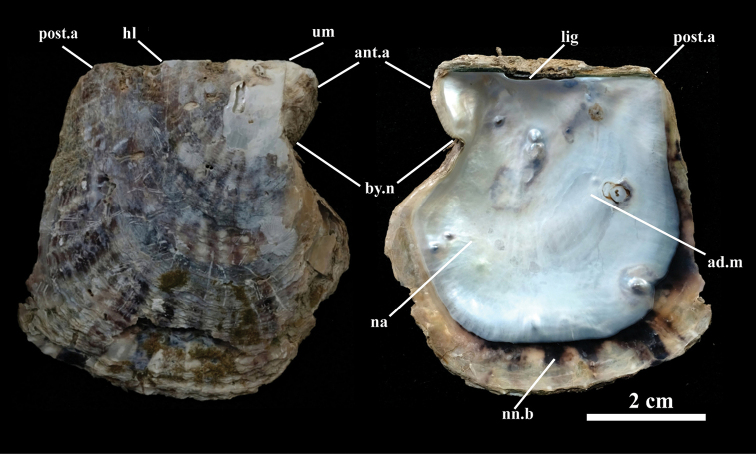
Right valve of holotype, KUMF.MOLL.1206 (NMR079), of *Pinctadaphuketensis* sp. nov., showing shell shape and structures. Abbreviations: ad.m, adductor muscle scar; ant.a, anterior auricle; by.n, byssal notch; hl, hinge line; lig, ligament; na, nacreous; nn.b, non-nacreous border; post.a, posterior auricle; um, umbo.

***Holotype***: KUMF.MOLL.1206 (NMR079) (Figs 1B, 2), 10 August 2021, collected by SCUBA diving. ***Paratypes***: two specimens, KUMF.MOLL.1204 (NMR077) (Fig. 1A) and KUMF.MOLL.1205 (NMR078) (Fig. 1C), 10 August 2021, collected by SCUBA diving. **Non-type material.** KUMF.MOLL.1201–KUMF.MOLL.1203, 5 February 2022, collected by SCUBA diving. All examined specimens were collected from the type locality by S. Somrup.

###### Diagnosis.

Shell is anteriorly oblique, inequilateral, laterally compressed, and subcircular to quadrate in outline. Byssal notch is small, narrow and slit-like. Hinge teeth are absent. Adductor muscle scar is kidney- or bean-shaped with the distal extremities of the posterior pedo-byssal retractor muscle scar inserted into the concavity on its anterior border. The non-nacreous border is relatively pale to transparent, with few dark brown or black blotches.

###### Description.

Holotype, KUMF.MOLL.1206 (NMR079), specimen is approximately 60.4 mm height, 53.1 mm length, 23.1 mm depth, and 22.9 mm width (Figs 1B, 2). Paratypes, KUMF.MOLL.1204 (NMR077) and KUMF.MOLL.1205 (NMR078), 53 and 78 mm height, 46 and 75 length mm, 8.3 and 43.7 mm depth, and 38.5 and 54 mm width (Fig. 1A, C), respectively.

The shell is rather thin and small. The shell height, which does not exceed 80 mm, is slightly greater than the length (Figs 1, 2). The shell convexity is moderate, with the left valve more convex than the right valve. The exterior surface of the shell (both valves) is typically dark greyish brown or green, crossed radially by alternating brownish black and lighter colored stripes. The non-nacreous margin has white porcelaneous patches, generally alternating with irregular, dark brown or black blotches and corresponding to the external coloration pattern. Growth processes on the outer surface of valves are small, flattened and brittle imbricating concentric scales which bear slender spines projecting radially towards the edge of the shell (Fig. 2). The posterior border is either small or absent from the posterior auricle. The dorsal margin is slightly curved and the umbonal area is low. Ridges on the back are high and obtuse, running from the umbo to the back end, with two faint secondary ridges (Fig. 2). The dark ligament is strong on the hinge line. Ligament is narrow, about ^3^/_4_ of hinge and elongation. The hinge line is straight, long and slightly shorter than the antero-posterior axis of the shell, with a ratio of 1:1.35 (Fig. 2). Hinge teeth are absent in the left valve and right valve (Fig. 2). The adductor muscle scar is kidney- or bean-shaped and clearly visible on the left valve. The right valve shows a larger attainment point scar on the shell. Scars on the back of the adductor muscle are very small. The anterior pedo-byssal retractor muscle scars are asymmetrical (Fig. 2). This structure is formed of individual byssal thread strands and extends ventrally and laterally from the base of the byssal groove to the short foot.

**Figure 3.**
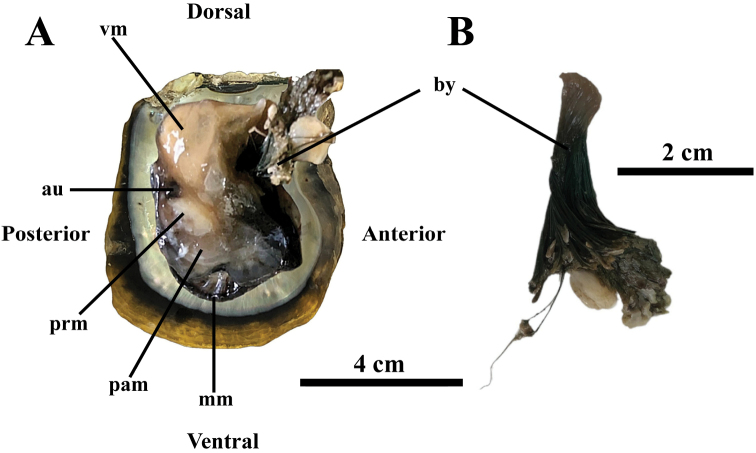
External view of the soft body parts of adult *Pinctadaphuketensis* sp. nov. **A** and close-up view of overall of byssus **B**. Scale bars: 4 cm (**A**); 2 cm (**B**). Abbreviations: au, auricle; by, byssus; mm, mantle margin; pam, posterior adductor muscle; prm, posterior pedo-byssal retractor muscle; vm, visceral mass.

For the soft body, the foot is a tongue-shaped organ located in the dorsal-anterior region of body, between the mouth and the byssus (Fig. 3A). Byssus threads are dark green with thickened stalk (Fig. 3B). Visceral mass is yellow and roughly half the size of its shell. It is ventral to the hinge and connected to the posterior adductor. The visceral mass contains digestive glands and gonad tissue. The heart is located posterior to the visceral mass, and consists of ventricle and auricles. Mantle margin is translucent dark, occupying most of the area between valves and extending from the hinge line (Fig. 3A). The color of the mantle margin is dark, corresponding to the internal non-nacreous shell, which has blotches or streaks of dark pigment. The posterior adductor muscle is large, kidney- or bean-like in outline and located slightly posterior to the visceral mass and attached to each valve (Fig. 3A). The posterior pedo-byssal retractor muscles are adjacent to the posterior adductor muscle and frequently inserted into its concave anterior border (Fig. 3A).

###### Etymology.

The specific epithet refers to the locality of Phuket Island, where this species was found.

###### Phylogenetic analyses.

Partial sequences of COI, 18S rDNA, ITS1, and ITS2 of recently collected *Pinctada* samples were successfully generated in this study. All sequences of *P.phuketensis* were identical for COI and 18S rDNA, and nearly identical for ITS1 (0.1–0.9% pairwise difference) and ITS2 (0.1–0.8% pairwise difference) but differed from sequences from other *Pinctada* species by at least 7% for COI, 0.2% for 18S rDNA, 2% for ITS1 and 1% for ITS2.

**Figure 4.**
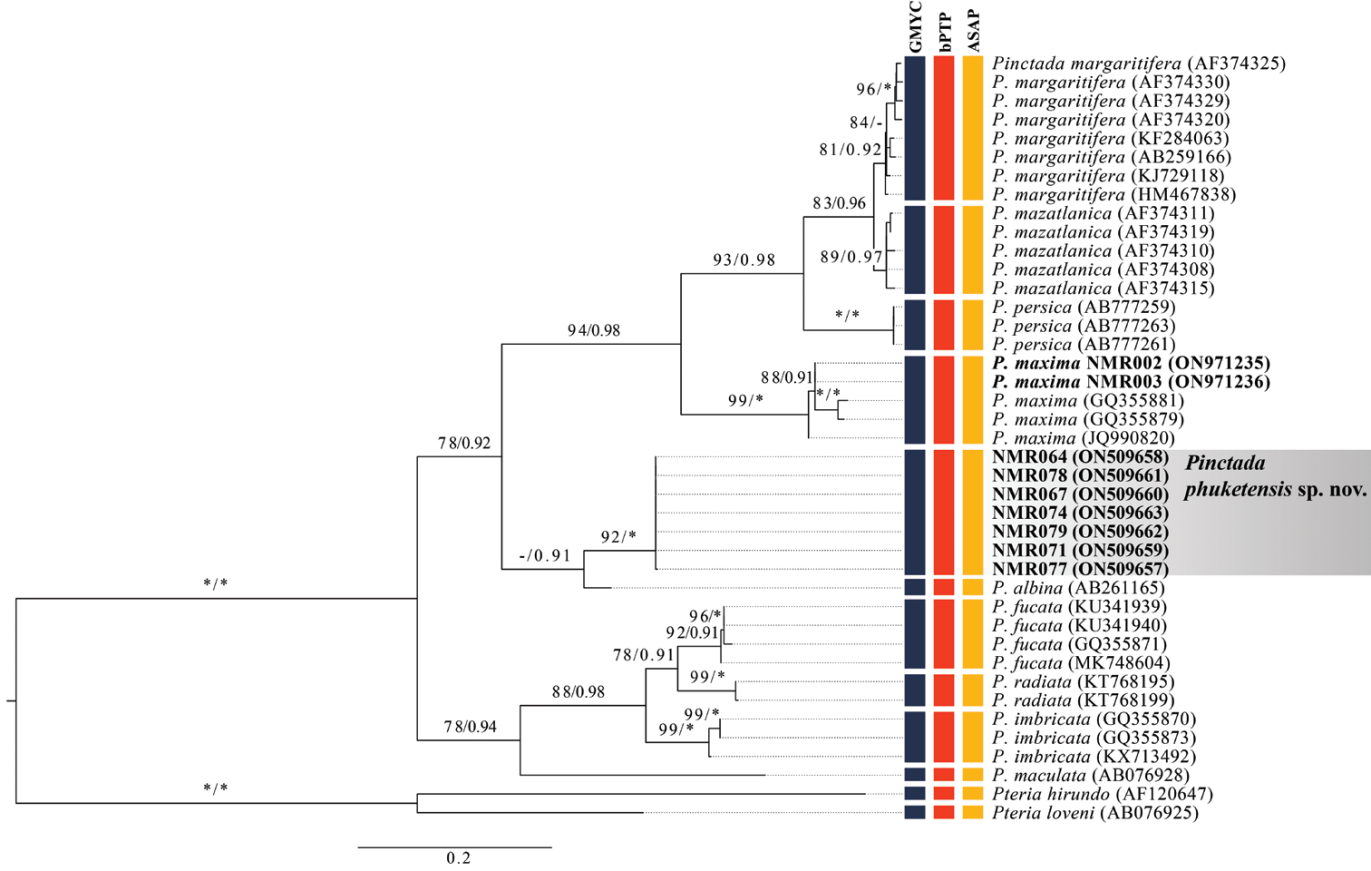
Maximum likelihood tree (-In L 4923.702) of partial COI sequences. Sequences of *Pinctada* specimens generated in this study are highlighted in bold. Support values are bootstrap/posterior probabilities. Asterisks indicate bootstrap (ML) value of 100% and posterior probability (BI) value of 1.00. Values < 50% ML bootstrap and < 0.90 posterior probability are not shown. GenBank accession numbers are given in parentheses. After the registration number or species name. Results of three species delimitation methods, namely GMYC model (blue column), bPTP (red column) and ASAP (yellow column), are indicated at the right edge of the tree.

The COI-based phylogenetic trees obtained by ML and BI analyses were topologically similar, and only the ML tree is shown (Fig. 4). The ML analyses indicated that all COI sequences of *P.phuketensis* sp. nov. formed a monophyletic group. *Pinctadaphuketensis* sp. nov. was clearly phylogenetically distinguished from other species with high support (ML = 96%, BI = 1.00), and was sister to *P.albina* from Japan (Fig. 4).

**Figure 5.**
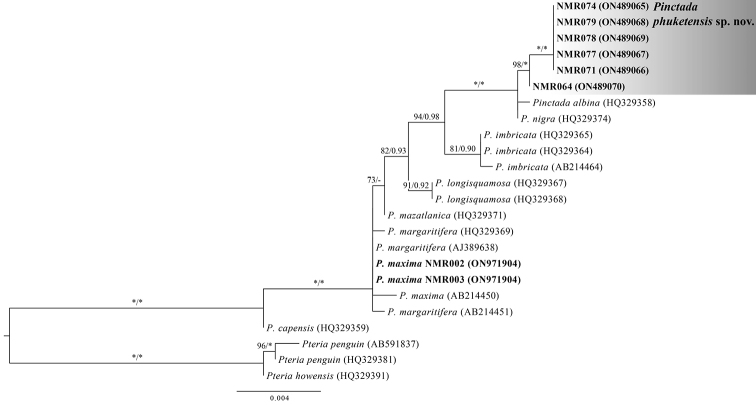
Maximum likelihood tree (-In L 3134.144) of 18S rDNA sequences. Sequences of *Pinctada* specimens generated in this study are highlighted in bold. Support values are bootstrap/posterior probabilities. Asterisks indicate bootstrap (ML) value of 100% and posterior probability (BI) value of 1.00. Values < 50% ML bootstrap and < 0.90 posterior probability are not shown. GenBank accession numbers are given in parentheses after the registration number or species name.

Additionally, phylogenetic analyses based on partial 18S rDNA sequences using ML and BI methods were highly congruent (Fig. 5). The ML tree supported the monophyly of *P.phukentensis* sp. nov., and a close relationship between *P.phuketensis* sp. nov. and two other *Pinctada* species, namely *P.albina* and *P.nigra*, with high bootstrap value (ML = 100%, BI = 1.00) (Fig. 5).

**Figure 6.**
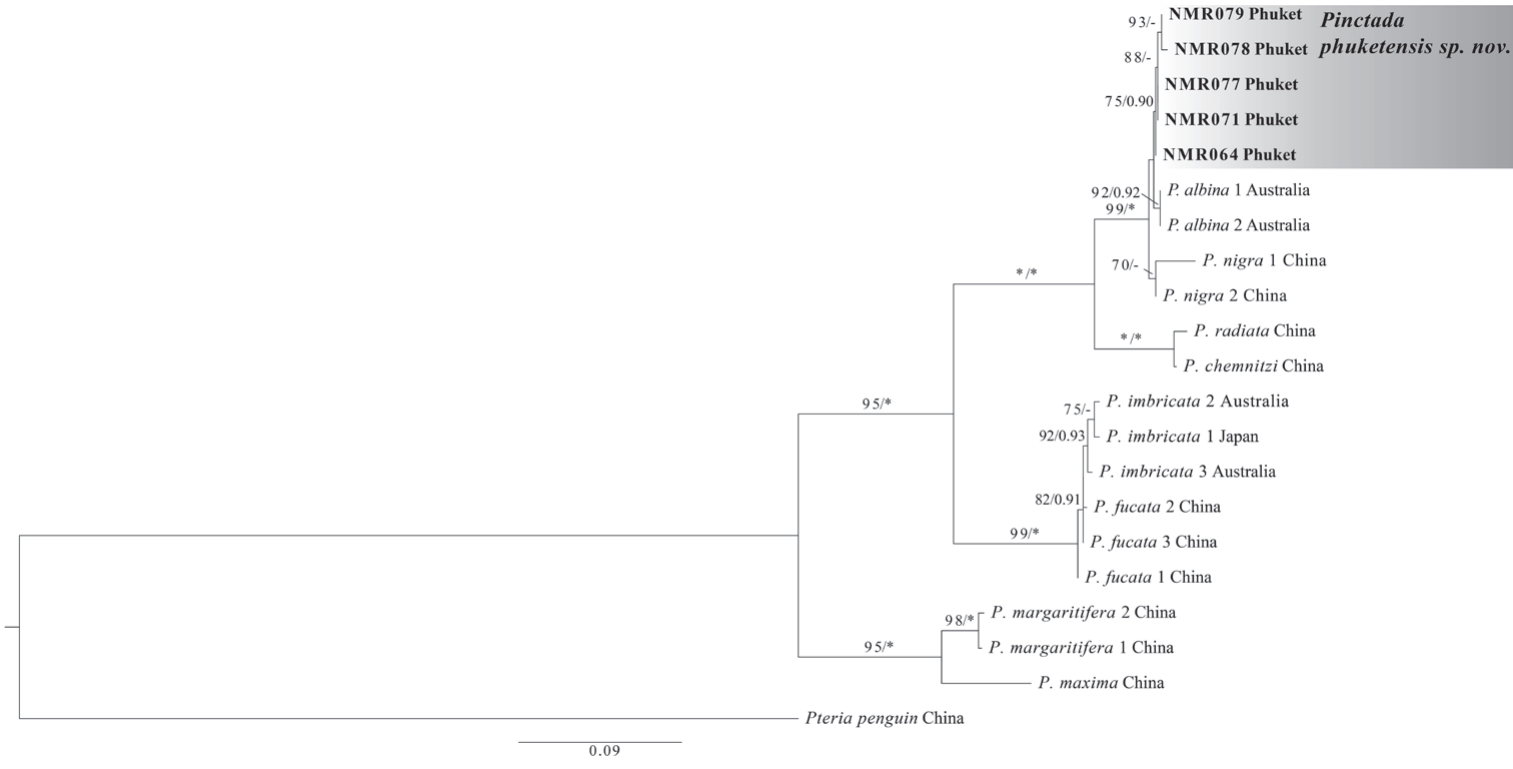
Maximum likelihood tree (-In L 4787.958) of combined ITS 1 and ITS 2 sequences. Sequences of *Pinctada* specimens generated in this study are highlighted in bold. Support values are bootstrap/posterior probabilities. Asterisks indicate bootstrap (ML) value of 100% and posterior probability (BI) value of 1.00. Values < 50% ML bootstrap and < 0.90 posterior probability are not shown.

Similarly, the phylogenetic relationships constructed by ML and BI methods based on the concatenated ITS1 and ITS 2 data set showed very similar topologies (Fig. 6). All sequences of *P.phukentensis* sp. nov. formed a well-supported clade, and this clade was grouped with *P.albina* from Australia and *P.nigra* from China with high support (ML = 99%, BI = 1.00) (Fig. 6).

### ﻿Species delimitation

Three different methods used for species delineation yielded the same number of putative species within *Pinctada* (Fig. 4). All methods, namely GMYC (L_GMYC_ = 138.8684 > L_0_ = 134.1486, *P* = 0.008), bPTP (acceptance rate = 0.14530, merge = 49971, split = 50029) and ASAP (*P* = 0.00004), clearly showed *P.phukentensis* sp. nov. to be distinct from its congeners (Fig. 4).

## ﻿Discussion

Our analyses using morphological and genetic data clearly distinguished the *Pinctada* samples recently collected from Dok Mai Island, Phuket Province, western coast of Thailand, from other *Pinctada* species in the region. Accordingly, these pearl oysters should be recognized as a new species, and we have named it as *Pinctadaphuketensis* sp. nov. This new species can be distinguished from other members of the genus by its smaller size, a subquadrate shell with moderately long ligament, slightly developed to undeveloped posterior auricle, the absence of hinge teeth, a pale to transparent non-nacreous margin with dark brown or black blotches, and brownish stripes on the external surface. A morphological comparison of *P.phuketensis* and some closely related species is presented in Table 1.

**Table 1.** Comparative morphology of *Pinctadaphuketensis* sp. nov. with other morphologically similar *Pinctada* species.

Character	*P.phuketensis* sp. nov.^1^	*P.albina* ^2,3^	*P.sugillata* ^2,3^	*P.chemnitzi* ^2^	*P.fucata* ^2,4,5^	*P.nigra* ^6^
Size	Small	Small	Small	Small	Small	Small
Shell shape	Slightly oblique	Slightly to moderately oblique	Very oblique	Moderately oblique and markedly inequivalve	Slightly oblique	Obliquely elongate
Anterior auricle	Small	Small	Small	Moderately to well developed	Larger	Small
Posterior auricle	Short and broadly rounded or absent	Small	Small	Larger	Short and broadly rounded	Large
Byssal notch	Small, narrow, slit-like	Broad	Moderately wide slit	Slit-like	Narrow and slit-like	n/a
Hinge teeth	Absent	Absent	Present	Present	Present	Present
External color	Green, yellow, brown, or partially continuous white blotches	White, possibly sun-bleached	Rayed or dark and white pattern to an evenly dark monochrome	Dull brownish, indistinctly rayed with paler shades	Red, brown, green and bronze	Green and dark
Nacre	White luster, nacreous and narrow black band on the non-nacreous border	Pale yellow throughout the nacre	Narrow black band on the non-nacreous border	Yellow throughout the nacre	White metallic luster, yellow, silver, gold, or pink tint	n/a

^1^This study, ^2^Hynd (1955), ^3^Hynd (1960), ^4^Takemura and Okutani (1958), ^5^Wada and Tëmkin (2008), ^6^Deng et al. (2019); n/a indicates information was not available.

Among the *Pinctada* species distributed in Southeast Asian waters, the new species of *P.phuketensis* morphologically resembles *P.fucata*, *P.nigra* and *P.albina*, but can be distinguished from these three species based on shell shape, hinge teeth and anterior/posterior auricles. Both *P.fucata* and *P.nigra* can be easily distinguished from *P.phuketensis* by having conspicuous hinge teeth. In addition, *P.fucata* can be separated from *P.phuketensis* by being larger in size and having a large and developed anterior auricle (Takemura and Okutani 1958; Colgan and Ponder 2002; Wada and Tëmkin 2008), while *P.nigra* clearly differs from our new species by exhibiting a large and developed posterior auricle and deep posterior sinus (Deng et al. 2019) (Table 1). On the other hand, *P albina* and *P.phuketensis* are similar in having no hinge teeth, but they can be differentiated by the characteristics of byssal notch and anterior border. *Pinctadaalbina* is distinguished from our new species by having a broad byssal notch and anterior border that projects well beyond the reference line (a line drawn at right angles to the hinge line through the byssal notch) (Hynd 1955) (Table 1).

Among the Indo-Pacific Ocean species, our new species, *P.phuketensis* closely resembles *P.sugillata* (Reeve, 1857) from Australia in having a weakly developed to undeveloped posterior ear and a nearly 1:1 ratio of the hinge line to the antero-posterior axis of the shell (Hynd 1955, 1960). However, these two species differ in shell shape (slightly oblique in *P.phuketensis* and very oblique in *P.sugillata*), hinge teeth (absent in *P.phuketensis* and present in *P.sugillata*) and byssal notch (narrow and slit-like in *P.phuketensis* and moderately wide and slit-like in *P.sugillata*) (Table 1; Hynd 1955, 1960). Unfortunately, genetic data for *P.sugillata* are not available. Further studies on *P.sugillata* will be useful for confirming that these two species are distinct.

Our phylogenetic analyses and species delimitation approach showed that *P.phuketensis* is genetically distinct from other described *Pinctada* species. While our observations indicated that our new species is morphologically similar to *P.fucata*, genetic analyses revealed the distant phylogenetic relationship between these two species, implying that morphological traits probably do not reflect their real evolutionary history. Additionally, our phylogenetic analyses showed that *P.phuketensis* is more closely related to *P.albina* and *P.nigra* than to *P.fucata*. We also found that phylogenetic relationships of some *Pinctada* species in this study had weak nodal support and were incompletely resolved. It is apparent that further work on *Pinctada* species based on combined data of different genetic markers and more expansive sampling from different geographic regions will uncover their diversity, phylogenetic relationships and evolutionary patterns.

## Supplementary Material

XML Treatment for Pinctada phuketensis

## Supplementary materials

Supplementary material 1 Table S1 Data type Docx file. Explanation note List of additional GenBank accession numbers for ITS1 and ITS2 sequences used in the present study. New sequences produced for this study are indicated in bold type. https://binary.pensoft.net/file/738226This dataset is made available under the Open Database License (http://opendatacommons.org/licenses/odbl/1.0/). The Open Database License (ODbL) is a license agreement intended to allow users to freely share, modify, and use this Dataset while maintaining this same freedom for others, provided that the original source and author(s) are credited.Supannee Somrup, Akkarasiri Sangsawang, Nichanun McMillan, Supanida Winitchai, Jitti Inthoncharoen, Shikai Liu, Narongrit Muangmai

Supplementary material 2 Table S2 Data type Docx file. Explanation note The best partitioning scheme and models of maximum likelihood (ML; IQ-TREE) and Bayesian inference (BI; MrBayes) methods for all datasets. https://binary.pensoft.net/file/738227This dataset is made available under the Open Database License (http://opendatacommons.org/licenses/odbl/1.0/). The Open Database License (ODbL) is a license agreement intended to allow users to freely share, modify, and use this Dataset while maintaining this same freedom for others, provided that the original source and author(s) are credited.Supannee Somrup, Akkarasiri Sangsawang, Nichanun McMillan, Supanida Winitchai, Jitti Inthoncharoen, Shikai Liu, Narongrit Muangmai
