# Supplementary material for: ﻿Pinctadaphuketensis sp. nov. (Bivalvia, Ostreida, Margaritidae), a new pearl oyster species from Phuket, western coast of Thailand
Source: Zookeys. 2022 Sep 2;1119:181–95. doi: 10.3897/zookeys.1119.87724 (PMC9848700; doi:10.3897/zookeys.1119.87724)
Supplement: Supplementary material 1 — Table S1 [file zookeys-1119-181_article-87724__-s001.docx]

**Table S1** List of additional GenBank accession numbers for ITS1 and ITS2 sequences used in the present study. New sequences produced for this study are indicated in bold type.

| Species | Sampling locality | GenBank accession number | |
| --- | --- | --- | --- |
|  |  | ITS 1 | ITS 2 |
| *Pinctada albina* 1 | Port Stephens, Australia | AY877498 | AY877508 |
| *P. albina* 2 | Port Stephens, Australia | AY877499 | - |
| *P. chemnitzi* | Daya Bay, Hainan, China | AY877496 | AY877509 |
| *P. fucata* 1 | Sanya Bay, Hainan, China | AY877523 | AY877586 |
| *P. fucata* 2 | Daya Bay, Hainan, China | AY877512 | AY877585 |
| *P. fucata* 3 | Beibu Bay, Hainan, China | AY877525 | AY877592 |
| *P. imbricata* 1 | Mie Prefecture, Japan | AY877577 | AY877612 |
| *P. imbricata* 2 | Port Stephens, Australia | AY877571 | AY877606 |
| *P. imbricata* 3 | Port Stephens, Australia | AY877569 | AY877609 |
| *P. phuketensis* NMR064 | Dok Mai Island, Phuket, Thailand | **ON489063** | **ON489071** |
| *P. phuketensis* NMR071 | Dok Mai Island, Phuket, Thailand | **ON489062** | **ON489075** |
| *P. phuketensis* NMR077 | Dok Mai Island, Phuket, Thailand | **ON489061** | **ON489074** |
| *P. phuketensis* NMR078 | Dok Mai Island, Phuket, Thailand | **ON489060** | **ON489073** |
| *P. phuketensis* NMR079 | Dok Mai Island, Phuket, Thailand | **ON489059** | **ON489072** |
| *P. margaritifera* 1 | Sanya, Hainan, China | AY877500 | AY877506 |
| *P. margaritifera* 2 | Sanya, Hainan, China | AY877502 | AY877507 |
| *P. maxima* | Sanya, Hainan, China | AY172345 | AY877504 |
| *P. nigra* 1 | Sanya, Hainan, China | AY192147 | AY282728 |
| *P. nigra* 2 | Sanya, Hainan, China | - | AY192714 |
| *P. radiata* | Sanya, Hainan, China | AY144603 | - |
| *Pteria penguin* | Sanya, Hainan, China | AY877503 | AY192715 |
