# Supplementary material for: ﻿Pinctadaphuketensis sp. nov. (Bivalvia, Ostreida, Margaritidae), a new pearl oyster species from Phuket, western coast of Thailand
Source: Zookeys. 2022 Sep 2;1119:181–95. doi: 10.3897/zookeys.1119.87724 (PMC9848700; doi:10.3897/zookeys.1119.87724)
Supplement: Supplementary material 2 — Table S2 [file zookeys-1119-181_article-87724__-s002.docx]

**Table S2** The best partitioning scheme and models of ML and BI methods for all datasets.

| **Method** | **Data set** | **Partition** | **Best model** |
| --- | --- | --- | --- |
| Maximum Likelihood (ML) – IQ | COI | Codon position 1 | TIM2+F+G4 |
|  |  | Codon position 2 | F81+F |
|  |  | Codon position 3 | GTR+I+G4 |
|  | 18S rDNA |  | TVM+F+I |
|  | ITS1 + ITS2 | ITS1 | GTR+F+G4 |
|  |  | ITS2 | GTR+F+I+G4 |
| Bayesian Inference (BI) - MrBayes | COI | Codon position 1 | TIM2+F+G4 |
|  |  | Codon position 2 | F81+F |
|  |  | Codon position 3 | GTR+F+I+G4 |
|  | 18S rDNA |  | K2P |
|  | ITS1 + ITS2 | ITS1 | TNe+G4 |
|  |  | ITS2 | TIM3+F+G4 |
